# Supplementary material for: Neurogenic vs. Myogenic Origin of Acquired Muscle Paralysis in Intensive Care Unit (ICU) Patients: Evaluation of Different Diagnostic Methods
Source: Diagnostics (Basel). 2020 Nov 18;10(11):966. doi: 10.3390/diagnostics10110966 (PMC7698781; doi:10.3390/diagnostics10110966)
Supplement: Supplementary file 1 [file diagnostics-10-00966-s001.pdf]

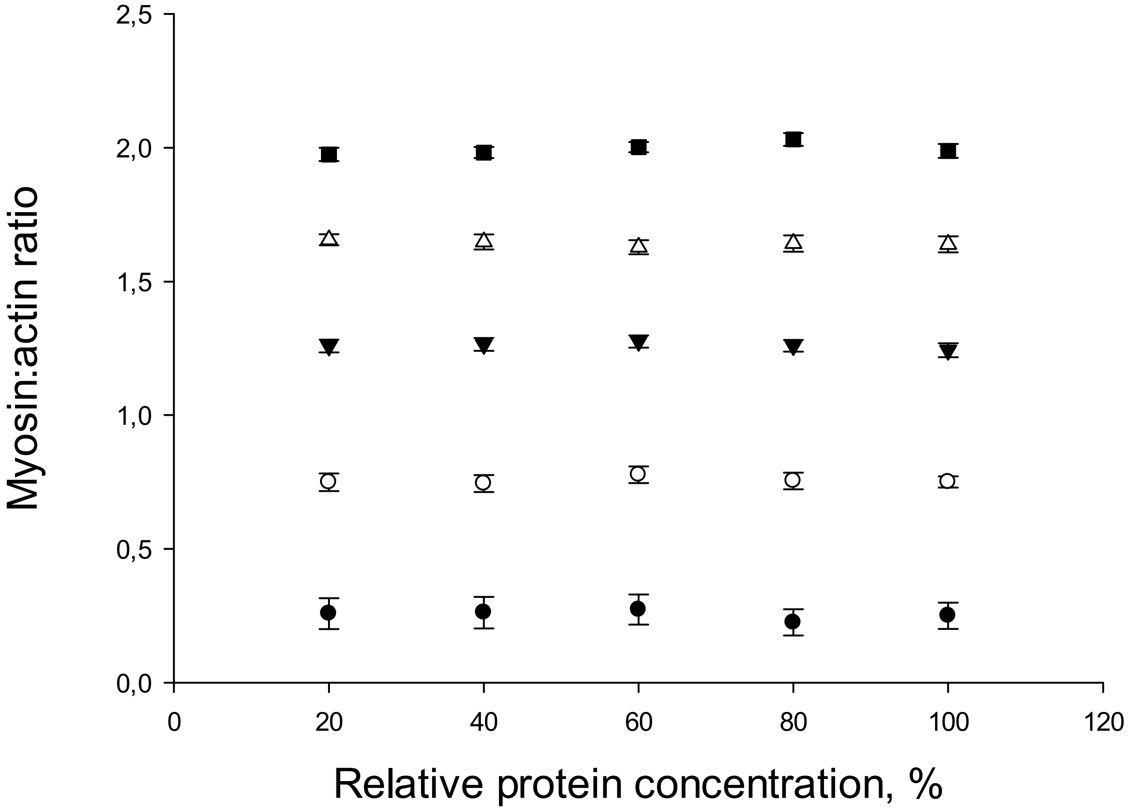


**Supplemental Figure S1.** M:A ratios measured at five different proteinconcentrations, i.e., the original proteinconcentration and dilutions at concetrations varying between 20 and 80% of the original concentration (100%). Protein concentrations has been compared between patients with a myosin ratio less than 0.49 (n=12), between 1.5 and less than 1 (n=31), between 1 and less than 1.5 (n=52), between 1.5 and less than 1.7 (n=26), and above 1.7 (n=47 and 24 of of them were healthy controls). According to one-way ANOVA, there was no significant difference in myosin:actin ratio at the different proteinconcentrations independent on the level of preferential myosin loss. Values are means ± S.E.M.

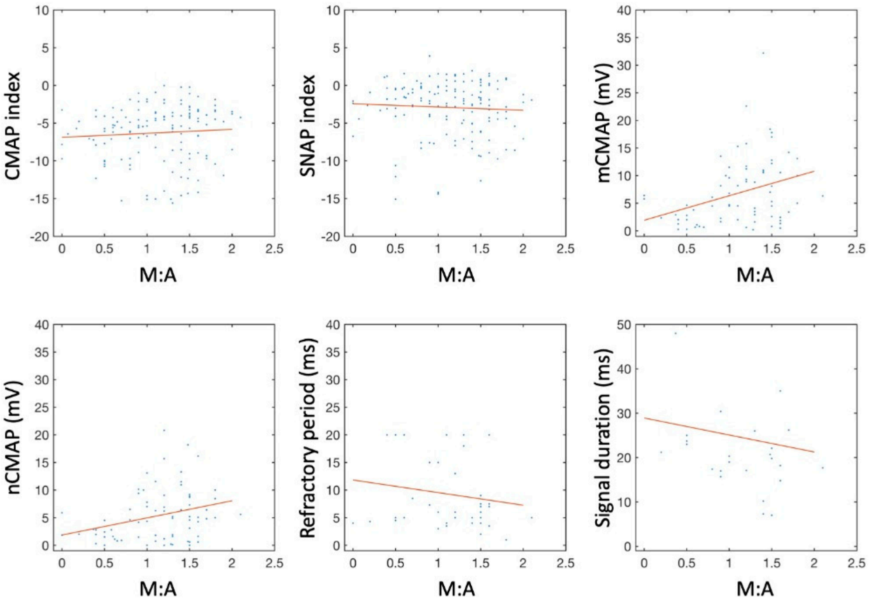


**Supplemental Figure S2.** Scatterplots of CMAP index, SNAP index, mCMAP amplitude, nCMAP amplitude, refractory period and signal duration vs. M:A ratio. Statistically significant ($p<0.01$) but weak correlations ($r=0.30-0.34$) were only observed between mCMAP and nCMAP amplitudes vs. M:A ratio.

**Supplemental table S1.** M:A ratios in healthy control subjects. An aging-related decline in the M:A ratio was observed in the oldest old. Therefore, the two oldest patients (87 and 94 years) were excluded and patients 85 years and younger were included for analysis in the study, since there was no statistically significant age-related decline in the control group in the 24-85 year age-range ( 24 men 24-79 years of age and 23 women, 24-83 years of age). 10, 20 and 25% indicate the number of healthy subjects with M:A ratio below the corresponding level.

| | **All subjects** | **≤85 years** |
|---|---|---|
| n | 55 | 47 |
| Mean | 1.93 | 1.99 |
| SD | 0.24 | 0.18 |
| < 2SD | 1.45 | 1.63 |
| 10% | 1.60 | 1.70 |
| 20% | 1.70 | 1.83 |
| 25% | 1.70 | 1.90 |
